# An effective method for quantification, visualization, and analysis of 3D cell shape during early embryogenesis

**DOI:** 10.1002/qub2.83

**Published:** 2024-12-20

**Authors:** Zelin Li, Zhaoke Huang, Jianfeng Cao, Guoye Guan, Zhongying Zhao, Hong Yan

**Affiliations:** ^1^ Department of Electrical Engineering City University of Hong Kong Hong Kong China; ^2^ Centre for Intelligent Multidimensional Data Analysis Limited Hong Kong China; ^3^ Center for Quantitative Biology Peking University Beijing Beijing China; ^4^ Department of Systems Biology Harvard Medical School Boston Massachusetts USA; ^5^ Department of Data Science Dana‐Farber Cancer Institute Boston Massachusetts USA; ^6^ Department of Biology Hong Kong Baptist University Hong Kong China; ^7^ State Key Laboratory of Environmental and Biological Analysis Hong Kong Baptist University Hong Kong China; ^8^ Present address: Department of Computer Science and Engineering Chinese University of Hong Kong Hong Kong China.

**Keywords:** C*aenorhabditis elegans* (*C. elegans*), cell shape quantification, eigen features (eigengrid, eigenharmonic & eigenspectrum), lineage analysis, morphological reproducibility, spherical harmonics (SPHARM)

## Abstract

Embryogenesis is the most basic process in developmental biology. Effectively and simply quantifying cell shape is challenging for the complex and dynamic 3D embryonic cells. Traditional descriptors such as volume, surface area, and mean curvature often fall short, providing only a global view and lacking in local detail and reconstruction capability. Addressing this, we introduce an effective integrated method, 3D Cell Shape Quantification (3DCSQ), for transforming digitized 3D cell shapes into analytical feature vectors, named *eigengrid* (*proposed grid descriptor like eigen value*), *eigenharmonic,* and *eigenspectrum*. We uniquely combine spherical grids, spherical harmonics, and principal component analysis for cell shape quantification. We demonstrate 3DCSQ’s effectiveness in recognizing cellular morphological phenotypes and clustering cells. Applied to *Caenorhabditis elegans* embryos of 29 living embryos from 4‐ to 350‐cell stages, 3DCSQ identifies and quantifies biologically reproducible cellular patterns including distinct skin cell deformations. We also provide automatically cell shape lineaging analysis program. This method not only systematizes cell shape description and evaluation but also monitors cell differentiation through shape changes, presenting an advancement in biological imaging and analysis.

## 1 INTRODUCTION

Embryogenesis, the developmental journey from a single zygote to a complex organism, is a cornerstone of developmental biology [1, 2, 3, 4, 5]. In model organisms, this process unfolds through a highly orchestrated sequence of cell division, enlargement, and differentiation exhibiting remarkable reproducibility across individuals [2, 6, 7, 8, 9]. The morphogenetic dynamics during embryogenesis are intricately linked to cell behaviors, including migration, proliferation, and differentiation governed by spatial and temporal changes in cell shape [9, 10, 11, 12, 13]. Despite significant advances in understanding the regulatory mechanisms of cell proliferation and migration in embryogenesis [14, 15, 16], a gap remains in quantitatively capturing and analyzing the dynamic morphological features of 3D cells. This study tried to close this gap by introducing an integrated method for the systematic quantification and analysis of cell shape during early embryogenesis.

*Caenorhabditis elegans* (*C. elegans*), a transparent nematode, serves as an exemplary model in developmental biology studies. Each cell in *C. elegans* is distinct and traceable, forming a comprehensive cell lineage and this lineage is characterized by consistent division timing, spatial positioning, cell volume, and cell fate across individuals [17], renowned for its invariant developmental patterns at the cellular level [1, 8, 18]. Traditionally, constructing a complete cell lineage morphological map of *C. elegans* from living embryos has been a labor‐intensive process, involving manual tracking of cell position, division, and fate. To streamline this process, half‐automated systems have been developed for tracking fluorescently labeled cell nuclei in *C. elegans* embryos. These systems, enhanced by deep learning, enable precise 3D cell shape segmentation [8, 9], thereby facilitating systematic and quantitative analyses of cell division, migration, and the complex dynamics of cellular structures in three dimensions.

More specifically, cells divide and differentiate into various lineages such as AB, MS, E, C, D, and P4 [1, 2, 8, 17], forming diverse cell types including intestinal, muscular, and skin cells. The orchestration of these cellular activities ensures the consistency in cell size, fate, and interaction underpinning the robustness of developmental patterns. The shape of a cell, influenced by both extrinsic forces and intrinsic cellular mechanisms, akin to the deformability observed in cancer cells, is a crucial determinant of cell fate and intercellular dynamics [4, 19]. Understanding these shape changes can unlock insights into cellular differentiation and morphogenesis. Metazoan embryonic development is marked by significant morphological transformations, exemplified by ABpl cells in *C. elegans* [9, 20]. A quantitative description of these cellular shape alterations offers a window into the complex interplay between cell shape and fate, enabling a deeper understanding of developmental biology [21, 22]. This research, therefore, emphasizes systematic and quantitative analysis of cell shape changes during key processes such as cell migration and differentiation, building on previous generative models of cell shape reconstruction.

Recent advancements in imaging technology have enabled the processes of dynamic time‐lapse fluorescence images, facilitating the 3D cell morphology map in developing organisms like *C. elegans* [9]. Some breakthroughs in this field has been the development of *CShaper* for *C. elegans* [9] and of ASTEC for Ascidian [4] which lays the groundwork for an in‐depth analysis and decoding of cell 3D shapes. However, the quantification and analysis of these shapes pose unique challenges. 3D structures in a comparable format is complex, as 3D shapes encompass more intricate details of deformation, movement, and cell interactions [8, 9, 23, 24]. Some feature extraction methods for 2D shapes, such as curvature and Fourier Transform [25], cannot keep enough information of 3D structures. Furthermore, description‐based features for 3D shapes [4, 8, 9] such as volume, surface area, and mean curvature [26] often fail to reconstruct the 3D shape, losing critical local information. To address this, Spherical Harmonics (SPHARM) modeling [22, 27], a technique for mapping 3D surfaces onto a unit sphere, emerges as a viable solution. This method yields a feature vector rich in both global and local shape information, providing a more comprehensive understanding of cellular morphology.

The exploration of how cell shape correlates with cell fate during *C. elegans* embryogenesis has been hindered by a scarcity of high‐quality data on living 3D cell shapes. Traditionally, the complexity of 3D shapes has led researchers to treat them as a series of 2D images rather than as holistic 3D entities [28]. This approach has limited the depth of understanding in 3D cellular morphologies. While there have been a few studies delving into 3D cell shape during embryogenesis, most research efforts have focused predominantly on segmentation and cell tracing in 3D confocal images [9, 24]. Although these studies have provided valuable methods for quantifying 3D shapes, a comprehensive and accurate quantification of the relationship between 3D cell shape and cell fate in *C. elegans* has yet to be established. This gap in knowledge underscores the need for more advanced and nuanced analytical models that can process and interpret the complex data associated with 3D cellular structures.

**FIGURE 1:**
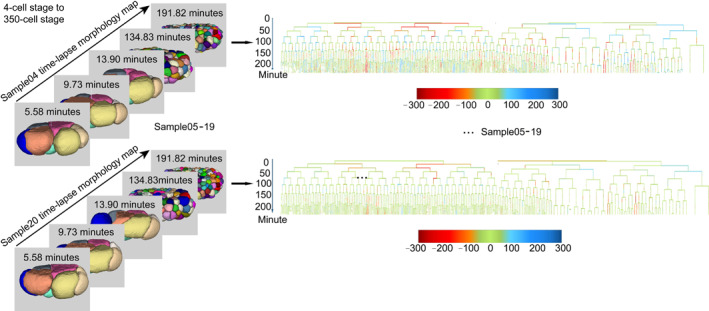
The lineage cell shape quantification. The digitized cellular 3D images 3DCSQ and the visualization of 17 compressed quantitative embryos as well as their lineaging cell shape visualization examples. Cells over time is quantified and visualized on cell lineaging during embryogenesis. The color bar represents the cell shape deforming level on *eigengrid* 1. 0 means no deformation and red and blue (negative and position 300) mean severe shape deformations.

We propose an effective 3D Cell Shape Quantification method, 3DCSQ, designed to quantitatively assess, visualize, and analyze cellular morphologies, particularly focusing on the embryogenesis of *C. elegans* from the 4‐ to 350‐cell stages (Figure 1). Here are our contributions.

- We integrate advanced algorithms, SPHARM, and widely‐used statistical method, PCA (principal component analysis), to produce a feature vector quantifying complicated cell shape. We introduce quantitative shape features, *eigengrid* (eigen spherical grid weights), *eigenharmonic* (eigen spherical harmonic coefficients), and *eigenspectrum* (eigen SPHARM spectrum coefficients) for studying interpretable growing patterns of cell shapes.
- With data provided in Refs. [8, 9] on *C. elegans* cell shapes and cell lineage at single‐cell resolution, 3DCSQ enables an in‐depth measurement of cellular morphologies. We also unveil patterns of shape reproducibility among individual embryos and identify cell clusters with shared fates.
- We have shown that the proposed quantitative features outperform other shape‐descriptors in reconstructing and recognizing skin cells. Extensive experiments utilizing these feature vectors assess various clustering algorithms, demonstrating that the proposed method can consistently reveal patterns of 3D cell shapes across embryos under various conditions (compressed and uncompressed worm embryos).
- 3DCSQ offers a visualizing on lineage approach, utilizing lineaging cell morphology analyses.

## 2 RESULTS

### 2.1 3DCSQ outline

We developed 3DCSQ for an automatic pipeline to analyze cell shape and hope to propose statistical features for live‐cell embryos. Step 1, 3DCSQ extracts the 2D grid according to the surface distance to the centroid as an image. Step 2, 3DCSQ transform these cell shape images to *eigengrid*, *eigenharmonic,* and *eigenspectrum* via PCA and SPHARM, fully automated in one python script. The calculation and algorithm are detailed stated in section materials and methods. Step 3, 3DCSQ provide qualitative lineage‐wise analyses and skin cells recognition (and other cells with relatively obvious deformations) in one package python code.

The 3DCSQ method adeptly demonstrates the reproducibility of cell shapes (normalized on volumes), efficiently identifies skin cells in compressed and uncompressed wild‐type embryos, and offers a comparative analysis of the performance of 3DCSQ and other shape descriptors in clustering tasks. Some terms should be illustrated. (1) Static shape feature is calculated for a cell at one time point and dynamic shape feature is the integration of a cell during its life cycle (appear and divide into 2 cells). (2) *eigengrid*, *eigenharmonic,* and *eigenspectrum* are referred to the weight vectors derived by PCA, containing only normalized shape information and excluding volume (size) information. (3) We use cell number as the main time scale; for correspondence of cell number and time, last 4‐cell stage is 0 min, 100‐cell is at ∼100 min, and 350‐cell is at ∼220 min (4) Average cell lineage is a filter‐like processing method for automated lineaging analysis.

### 2.2 Shape reproducibility of *C. elegans*

**FIGURE 2:**
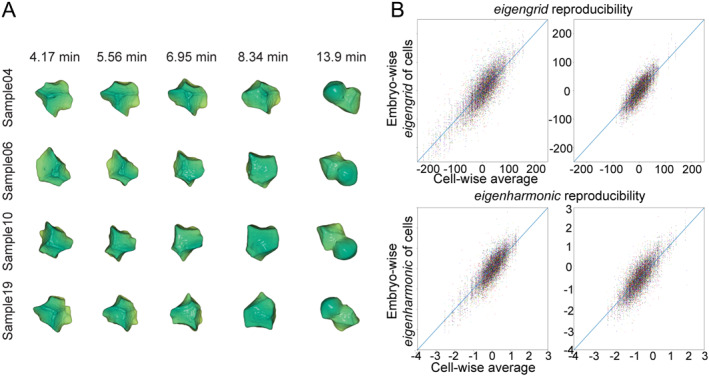
Qualitative and quantitative cell shape reproducibility analysis. (A) Qualitative cell developing shapes: similar developmental cell shapes across embryos, with consistent morphological reproducibility. The directly average shape from embryos has no visualized morphological features without registration but can be quantitatively distinguished. (B) Quantitative morphology and reproducibility assessment: illustrating the reproducibility of cell shapes (1218 cells from an average cell lineage tree of 17 embryos over 220 min of embryogenesis) through the analysis of *eigengrid* 1 and 3 (2 is excluded here) and *eigenharmonic* 1 and 2, which account for 27% and 10.4%, 17.2% and 14.3% of morphological deformation information, respectively. Each color in the graph represents an individual embryo, with points plotted according to dynamic features derived from *eigengrids* or *eigenharmonic*.

Cell shape deformations are studied via their shape changing (normalized by volume) to exclude the influences from cell size. Cell fate, volume, and surface are reproducible (in certain range) in Refs. [9, 17]. A closer look at cell morphology, we found that the cell shape is reproducible and invariant among individuals, such as certain cell fates [17], cell positions [29], volumes, and surface areas [9]. Thus, cell shape is invariant in a small dynamic range during its early embryogenesis (before the 350‐cell stage). The reproducibility study involves the dynamic shape features of cells, derived from entire cell cycles, and the experiment is conducted on 17 compressed and 8 uncompressed wild‐type embryos [8, 9]. We qualitatively demonstrated that cell shapes change in a similar manner at identical developmental stages/timings among all embryo (compressed samples are shown in Figure 2A); the early cell ABpl, a cell with remarkable deformations, is used for showing the cell reproducibility. We found that, in different samples but at the same time, ABpl has the same degree of protrusions, depressions, and deformation.

Only qualitative reproducibility for one cell is not convincing, we showcased the quantitative reproducibility for all cells in total 29 embryos. We select the eigen features with most morphological information (explained variance in Tables S3 and S4), whose variance ratio are 27%, 10.4%, 17.2%, and 14.3%, respectively (*eigengrid* 1, *eigengrid* 3, *eigenharmonic* 1, and *eigenharmonic* 2). Even embryos and cells always have their orientation, the small detailed shape structures with rotating can be captured in the proposed features, *eigengrid*, *eigenharmonic,* and *eigenspectrum*. We mainly use the former 2 features which can do cell shape reconstruction via several parameters. We analyze 350 cell shapes with a whole lifespan and fit the data to their average (Figure 2B) with *eigengrid* 1 and 3 (2 is excluded here for its unobvious phenomenon) and *eigenharmonic* 1 and 2. The morphologically importance levels of eigen features are derived from the explained variance of calculation in PCA (Tables S3 and S4). The reproducibility in uncompressed eight embryos are obvious as well (Figures S1 and S2), from which we can conclude that cell shape is invariant in both compressed and uncompressed embryos/individuals. Same cells distribute close, showing the fact that a cell always keeps in a normalized similar shape during its developmental period, which also indicates a high accuracy and reproducibility in cell shape control during embryogenesis. Meanwhile, *eigengrid* and *eigenharmonic* are available in Tables S1 and S2. Notably, for 94% of the 1218 cells, *eigengrid* 1 deformed under 25% of highest value (50 of 200) across individual compressed embryos, indicating the reproducible cell deformations. *eigenharmonic* 1, 90% of cells also deform within 25% (0.5 of 2). This is consistent with what we saw in Figure 2B, and further demonstrates a high degree of precision and consistency in cell shape regulation during embryogenesis.

Conclusively, the observed linear relationships between individual and average cell shapes suggest that *C. elegans* employs regulatory mechanisms to maintain highly invariant cellular shapes within small deformations, complementing constraints on positions, volumes, surfaces, and division times, thereby ensuring a controlled and stable developmental environment. The cell shape reproducibility constraints every cell’s growing environments, which is very important to keep the cell–cell communication network in a programed structure, to keep the normal embryonic development.

### 2.3 Recognition of skin cells

Our analysis of cell shapes in *C. elegans* successfully identifies skin cells using the weight of the first *eigengrid*. This technique is particularly effective during the 200‐ to 350‐cell stage (150 to 220 min phase) of embryogenesis. In *C. elegans*, cell lineages, including the skin cells (ABa and ABp sublineages of the posterior two granddaughters, anterior two granddaughters, and the C sublineage daughters), are traceable and named systematically. For visualization, these cells can be distinctly marked in the average cell lineage tree.

**FIGURE 3:**
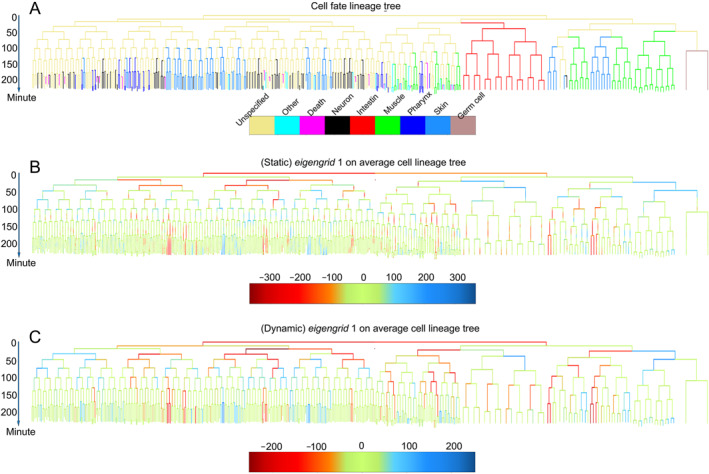
Lineage eigengrid cell shape analysis. (A) Cell fate lineage tree: showcases the cell lineage tree with cell fate annotated, providing a visualization of cell division and differentiation stages up to the 350‐cell stage. (B) and (C) Red indicating positive and blue indicating negative weights of *eigengrid* 1. (B) Presents static feature vector is the weight of the first *eigengrid* in every minute in average cell lineage tree. (C) Shows dynamic feature vector is the average of weights of the first *eigengrid* of the same cell during cell cycle defined before.

To demonstrate the lineage analysis, we show the fate‐wise cells colored with their eventual fates in Figure 3A. Analysis of 17 embryos revealed that skin cells typically exhibit higher absolute values in *eigengrid* 1, suggesting deformation skin to the shape of the first *eigengrid*, resembling a funnel. In the average cell lineage tree, the quantification of each cell at each minute (static feature) represents the average of these weights among individuals (Figure 3B,C). At period after 100 min of embryogenesis, cells in the lineage tree with *eigengrid* 1 exceeding fixed values 5 and 120 (absolute weights of *eigenharmonic* 1 over fixed values 5 and 1.4) are identified as skin cells. The color, blue and red in Figure 3, representing the depressions and protrusions on cell surface, corresponds to the degree of deformation in *eigengrid* 1. The observed large values in the *eigengrid* 1 suggest the horizontal ring depressions deformation at the middle of cells that persist throughout the cell cycle, including during mitosis division and differentiation. This deformation is likely a key factor in skin cell formation. Notably, most cell differentiations occur after the 100‐cell stage (100 min), allowing for effective recognition of skin cells in the lineage tree post this period. Using the dynamic feature (*eigengrid* 1) and a threshold of fixed value 5 (Figure 3C, Tables 1 and 2), 120, in Table 3, the classification precision for skin cells after 100 min development reaches 100% in the cell lineage tree analysis. While 3DCSQ may not identify all skin cells, the ones recognized are definitively skin cells. However, the best threshold is fixed value 3 and 110 which could keep the better accuracy with both moderate/not bad precision and recall.

**TABLE 1:** The silhouette score of different static feature vectors with different clustering methods.

Clustering method	*eigengrid*	*eigenharmonic*	*eigenspectrum*
K‐means	0.1	0.1	0.38
Ward	0.03	0.04	0.39
Average	0.23	0.25	**0.61**
Maximum	0.03	0.02	0.5
Single	0.28	0.29	0.55

*Note*: The ward, average, maximum and single are achical clustering algorithms’ parameters. Higher scores demonstrate better ability of clustering (classifying) cells by shape into different groups. Bold value demonstrate the best clustering way to do classify 3D cell shape. Underline value is the second best.

This application implied that it is possible to classify different tissue cells even in the early embryogenesis. Skin cells consist of the relatively obvious shape deformations, so they could be recognized by this explicit and simple way.

### 2.4 Cell shape feature vector clustering

Clustering is a way to do unsupervised learning to find out objects with common properties and patterns. K‐means, DBSCAN, and hierarchical clustering algorithms are applied on dynamic and static feature vectors proposed in section 3DCSQ method. The silhouette coefficient of DBSCAN results is inflated, and for fairness, we compare the performance of K‐means and hierarchical clustering.

The silhouette coefficients indicate the viability of clustering cells based on shape. The bold value means the best result (best way to do unsupervised learning/clustering) and the underlined value means the second best way to do clustering. In our analysis, reflected in (Tables 1 and 2), reveals that the *eigenspectrum* feature vector, combined with average linkage hierarchical clustering, provides the most effective clustering and potential classification of different cells. This finding underscores the importance of using rotation‐invariant features for clustering tasks, highlighting the potency of *eigenspectrum* features in future cell shape analyses. The 3DCSQ’s capability to extract these *eigenspectrum* features obviates the need for developing new feature extraction methods, streamlining future studies and experiments in this domain. Additionally, even *eigenspectrum* has the highest silhouette coefficients, it has no ability to reconstruct the original shape. Thus, we need to know the keys of different tasks and choose the proper one or several *eigen* features.

**TABLE 2:** The silhouette score of different dynamic feature vectors with different clustering methods.

Clustering method	*eigengrid*	*eigenharmonic*	*eigenspectrum*
K‐means	0.11	0.1	0.37
Ward	0.08	0.07	0.32
Average	0.14	0.14	**0.39**
Maximum	0.06	0.06	0.34
Single	0.14	0.15	0.34

*Note*: The ward, average, maximum and single are hierarchical clustering algorithms’ parameters. Higher scores demonstrate better ability of clustering (classifying) cells by shape into different groups. Respectively, bold and underline values represent the best and second best way to classify 3D cell shape.

**TABLE 3:** The precision and recall of different methods.

Feature name	Threshold of the features	Fixed value 1	Fixed value 2	Fixed value 3	Fixed value 4	Fixed value 5
*eigengrid* 1	Precision	75%	77.78%	87.5%	92.86%	100%
	Recall	68.2%	66.7%	66.7%	65%	65%
*eigenharmonic* 1	Precision	60%	75%	81.3%	90.9%	100%
	Recall	68.2%	68.2%	65%	58.8%	58.8%

## 3 DISCUSSION

The 3DCSQ methodology proposed 3 shape features, *eigengrid*, *eigenharmonic*, and *eigenspectrum*, from single cells, providing available cell shape quantification in lineaging analysis (automated visualization and statistical). These features enabled effective distinction and clustering of cellular shapes, exhibiting high reconstruction accuracy.

Biologically, 3DCSQ validated the reproducibility of cell shapes among *C. elegans* individuals. For instance, the similarity in shapes of cells like ABpl across individuals underscores this reproducibility. 3DCSQ’s ability to establish shape invariance across identical cells of 25 wild‐type embryos, provides a robust measure of shape consistency. Another notable application of 3DCSQ is in identifying skin cells with high precision during the 150 to 220 min embryogenesis period, up to the 350‐cell stage. This methodological advancement enables two critical progressions: First, the classification of cells’ eventual fate by their normalized cell shape eigen features, and second, the integration of cell lineage information with cellular shape analysis using dynamic features, automatically. Comparative analysis of clustering performances of *eigengrid*, *eigenharmonic,* and *eigenspectrum* demonstrates the superiority of the *eigenspectrum* due to its rotation‐invariant properties. This finding offers valuable insights for future embryogenesis studies at the cellular level.

Given that existing shape quantification studies are often unable to cater to cellular‐level developmental processes, the introduction of 3DCSQ marks a significant stride in quantifying cell shapes specifically for early embryogenesis studies. This approach promises to enhance research focused on the cellular dynamics during developmental stages.

## 4 CONCLUSION

This study introduces an automated and effective 3DCSQ method, focusing on representation, quantification, and pattern recognition at the cellular level during embryogenesis. The method, abbreviated as 3DCSQ, effectively analyzes feature vectors to discern biological patterns, specifically identifying skin cells in the average cell lineage tree of 350‐cell stage *C. elegans* embryos. Despite the technical challenges in pattern finding within cell shapes, 3DCSQ demonstrates proficiency in reproducing patterns, recognizing skin cells, and visualizing the mean cell lineage tree. In its pursuit of large‐scale cell shape analysis and the exploration of optimized feature vectors for cell clustering, 3DCSQ reveals the superior clustering efficiency of the energy spectrum, assessed through K‐means and hierarchical clustering algorithms.

Future work will focus on validating and exploring cell shape patterns with higher silhouette scores, employing deep learning to predict cellular movement using 3D cell shape and positional data, and constructing mechanical models of single cells during embryogenesis. Additionally, incorporating cell–cell contact, cell position, and cell movement into the quantification model—factors crucial in embryogenesis—will enhance the effectiveness of feature vectors in future iterations of 3DCSQ.

## 5 MATERIALS AND METHODS

Additionally, 3DCSQ assigns feature vectors to each cell shape at every developmental stage, termed as “static features” herein. Furthermore, it considers cells during a cell cycle as a singular entity, averaging their feature vectors to generate “dynamic features”.

**FIGURE 4:**
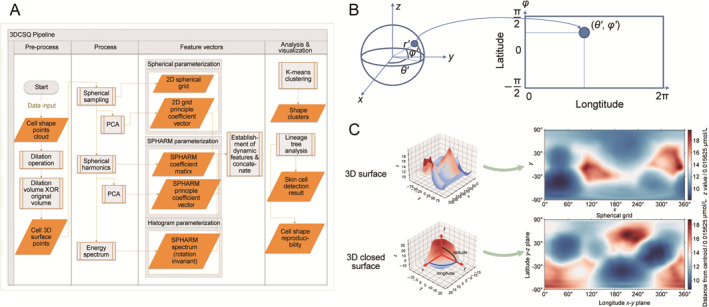
The detailed computational workflow and dataflow of 3DCSQ. The chart (A) describes how 3DCSQ performs morphological operations, samples, extracts feature vectors, establishes dynamic features, clusters and analyzes cell lineage tree. The eigengrid Weight Vector (weights of *eigengrid*) is the 2D Spherical Grid transformed to low dimensional vector by PCA. The eigenharmonic Weight Vector (weights of *eigenharmonic*) is the SPHARM Coefficient Vector transformed to a low dimensional space by PCA. The Energy Spectrum is the power sum of SPHARM coefficients of all orders of every degree. (B) Presents the way of sampling on spherical object to get 2D grid for this object. Along latitude and longitude axis, the local information and global information of the object would be collected into a rectangular grid. A 2D spherical grid in (C) illustrates how 3DCSQ mapped the points homogeneously a 3D closed surface. This is similar to plotting an area contour line. For a spherical grid, the *x*‐axis is the longitude (azimuthal angle) range from 0° to 360° and the *y*‐axis is the co‐latitude (polar angle) from −90 to 90° defined in spherical coordinate. Two cells are shown here from Sample06 during its embryogenesis.

3DCSQ provides a feasible method, illustrated in Figure 4A, to quantify, cluster and analyze cell shape. In the 3DCSQ workflow, the point clouds of digitized 3D cell shape are transformed to a group of surface points in preprocessing step (Figure 4B). These surface points of a cell are then transformed to a feature vector (Figure 4C). Given visualization method of cell lineage tree is provided to facilitate biologists finding regulatory patterns of 3D cell shape during embryogenesis of *C. elegans*. The input of 3DCSQ is a set of point cloud of a cell in an embryo while the output is cell shape feature vectors, quantitative cell lineage tree, and statistical results. Analysis and visualization by 3DCSQ enable researchers to plot and analyze cell shape deformation and cell fate across individuals during embryogenesis.

### 5.1 Data, cell morphology, and preprocessing

In this work, 3DCSQ is built with *C. elegans* 4D images (3D cell objects + time for live embryos). 2D cell images stacked along the *z*‐axis are processed to give 3D digitized (segmented) data in *CShaper* [9]. More information can be found in the supplementary files.

With each embryo digitized as a 3D array, based on 3D time‐lapse segmentation, pixels in the region of each cell are labeled with the same number (every cell is traced). Therefore, the region of a cell can be queried by its name. Let *D* be the point cloud of one digitized 3D cell shape, then:

$$D=A⊕B=z\in E|\left(B^{s}\right)z\cap A\neq \emptyset$$

where *A* is the point set representing a cell, *B* is a 3 × 3 × 3 structure element, *E* is a Euclidean space including *A* and $B^{s}=x\in E|-x\in B$. The surface points set *S* of a cell can be expressed in terms of a logical disjunction between the original point set *A* and dilation point set *D*.

### 5.2 Establishment of effective cell shape feature vector

Unlike other conventional 3D shape descriptors, spherical grid, SPHARM, and Energy Spectrum are generative models—the models can reconstruct the original 3D shape with sufficient local information. Thus, each 3D shape is transformed to a feature vector in a feature space, and this feature vector can be transformed back to the 3D original shape (the accuracy of restoration depends on the number of the feature space dimension). The effectiveness of quantification of 3D shape can be verified by comparing the original cell shape and the reconstructed cell shape. In addition, during the cell cycle, if a cell changes continuously in Euclidean space, its feature vector would change continuously in feature space. That is why 3DCSQ can do interpolation for lost cells (segmentation failed) during the cell cycle to predict 3D cell shape. Accordingly, the features of the same cell at every minute on average can represent this cell’s shape features during its cell cycle, which are named dynamic features in the following.

As the first part of the process step (Figure 4B), 3DCSQ constructs a 2D spherical grid (53 × 105) for every 3D digitized cell surface by sampling points on the 3D cell surface. To build a spherical grid $F_{\text{grid}}\left(\theta ,\phi \right)$ (similar to a 2D grayscale figure), points that are distributed unevenly on cell surface are sampled homogeneously to a 2D grid by the polar angle *θ* and azimuthal angle *ϕ* using the nearest interpolation on the unit sphere (Figure 4B). Only in this way can the 2D grid be formed to represent the 3D shape (Figure 4C). To eliminate the influence of volume, the 2D grids are normalized by their volume corresponding coefficient; the distance from the centroid, $r^{\prime }$, would be $\hat{r^{\prime }}=r^{\prime }/\left(\sqrt{3}V\right)$. Thus, one of the rectangular and comparable feature vectors are available, allowing reconstruction, comparison and calculation on 3D cell shape. The normalized 2D grids of all cells (totally 378,434 cells of compressed 17 embryos from 4‐ to 350‐cell stage) can be found in the supplementary files. To imagine a world map or an undulating sphere (Figure 4C), every point on this sphere is part of a sag and a crest, which means they are not in the same position along the centroid of the 3D shape. The distance of a point from the cell’s centroid is the value of that point on the 2D spherical grid. Although equispaced sampling will lead to oversampling at the north and south poles, this representational method can extract most features locally and globally of a cell shape to allow the transformation from a 3D shape to a 2D grid.

**FIGURE 5:**
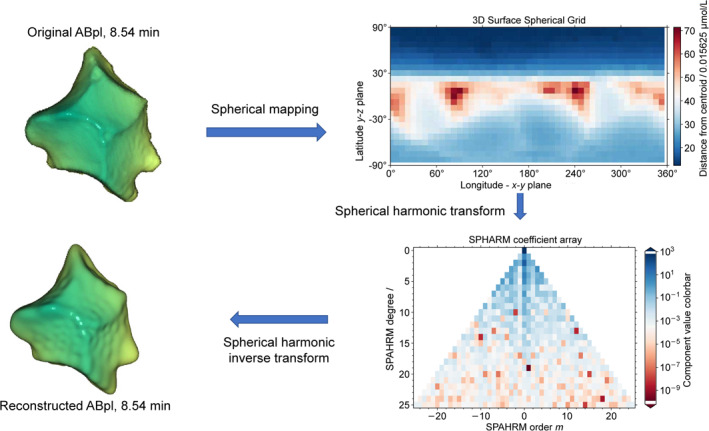
Real living cell shape representation in 3DCSQ. SPHARM transform of a 3D surface (ABpl cell at 8.54 min in Sample05 embryo) to a coefficient grid. Each coefficient represents the weight of its spherical harmonic shape in the original shape. The inverse SPHARM transformation reconstructs the 3D surface with 676 coefficients. The resilience of the SPHARM feature vector appears to be such that the original shape is clearly expressed.

The Spherical harmonics (SPHARM) is a form of Fourier series transformation (Figure 5), also known as an extended Fourier spherical harmonic function or Laplace’s spherical harmonics. This transformation can be applied to parameterize a 3D cell shape using Driscoll and Healy’s sampling theorem [30], especially a spherical object. To find the surface function, the cell surface can be expanded to a sum of SPHARM surface functions. This is similar to periodic functions defined on a curve that can be expressed as a sum of different order sines and cosine functions via Fourier Series. Unlike Fourier Series, another set of 3D orthogonal functions, $Y_{l}^{m}\left(\theta ,\phi \right)$, is used. These can be viewed as the angular portion of solutions to Laplace’s equation in 3D closed surface [26]. After mapping all the sample points, r(θ,ϕ)=(x(θ,ϕ),y(θ,ϕ),z(θ,ϕ)), on the cell surface the surface equation f(θ,ϕ) represents the cell surface in the spherical coordinate system. By transforming a 3D surface to SPAHRM coefficient vector, the definition of the Laplace spherical harmonics $Y_{l}^{m}:S^{2}\to C,Y_{l}^{m}$ forms a complete set of orthonormal basic functions in a Hilbert space using SHTools [31]. Thus, on the surface of a unit sphere $S^{2}$, the surface equation f: $S^{2}\to C$ is expanded and represented as follows:

$$f\left(\theta ,\phi \right)=\sum_{l=0}^{L}\sum_{m=-l}^{l}f_{l}^{m}Y_{l}^{m}\left(\theta ,\phi \right),$$

where the $f_{l}^{m}$ refers to

$$f_{m}^{l}=\int_{\Omega }f_{l}^{m}Y_{l}^{m}\left(\theta ,\phi \right)d\Omega$$

$$=\int_{0}^{2\pi }d\phi \int_{0}^{\pi }d\theta \;\sin\;\theta f\left(\theta ,\phi \right)Y_{l\left(\theta ,\phi \right)}^{m}.$$

This is the SPHARM calculation method while $f_{m}^{l}$ is calculated from a Fourier integral function rather than a standard least‐squares estimation [26, 27]. The integral equation above gives more accurate SPHARM coefficients from the sampling points on the cell surface. The SPAHRM orthogonal basic function is as follows:

$$Y_{l}^{m}\left(\theta ,\phi \right)=\sqrt{\frac{2l+1\left(l-m\right)!}{4\pi \left(l+m\right)!}}P_{l}^{m}\left(\cos\;\theta \right)e^{\text{im}\phi },$$

where $P_{l}^{m}\left(\cos\;\theta \right)$ are the associated Legendre polynomials defined by the differential equation:

$$P_{l}^{m}\left(\omega \right)=\frac{{\left(-1\right)}^{m}}{2^{l}!}{{\left(1-\omega^{2}\right)}^{m/2}\frac{d^{l+m}}{d\omega^{l+m}}\left(\omega^{2}-1\right)}^{l}.$$

This equation enables the SPHARM transformation, using $Y_{l}^{m}\left(\theta ,\phi \right)$ to represent the surface function and surface point distribution. Considering *l* as the SPHARM degree and order *m*, a 3D cell object would be represented by a vector $F_{\text{sp}h\text{arm}}\left(l,m\right)$:

$$F_{s\text{pa}h\text{rm}}={\left[f_{0}^{0},f_{1}^{-1},f_{1}^{0},f_{1}^{1},\cdot \cdot \cdot ,f_{L}^{-L},f_{L}^{-\left(L-1\right)},\cdot \cdot \cdot ,f_{L}^{\left(L-1\right)},f_{L}^{L}\right]}^{T},$$

where $F_{\text{sp}h\text{arm}}\left(l,m\right)$ represents a single cell shape in one sample, L means the max degree used in the SPHARM transform. This is the SPHARM coefficient vector. The reconstructed cell is compared with the original cell in (Figure 5) using 676 (262) SPHARM coefficients.

Energy spectrum $F_{\text{spectrum}}\left(l\right)$, a rotation‐invariant shape feature vector is defined by SPHARM feature vector $F_{\text{sp}h\text{arm}}\left(l,m\right)$. $F_{\text{spectrum}}$ is computed and summed up for all orders *m* of each degree *l*. $F_{\text{spectrum}}$ represents the cell shape deformation energy in SPHARM each degree *l* and allows cell shape to be analyzed with rotation and translation invariant property. 3DCSQ uses the power spectrum $F_{\text{spectrum}}\left(l\right)$ which can be calculated by $F_{\text{spectrum}}\left(l\right)=\sum_{m=-l}^{l}f_{l}^{m^{2}}$.

**FIGURE 6:**
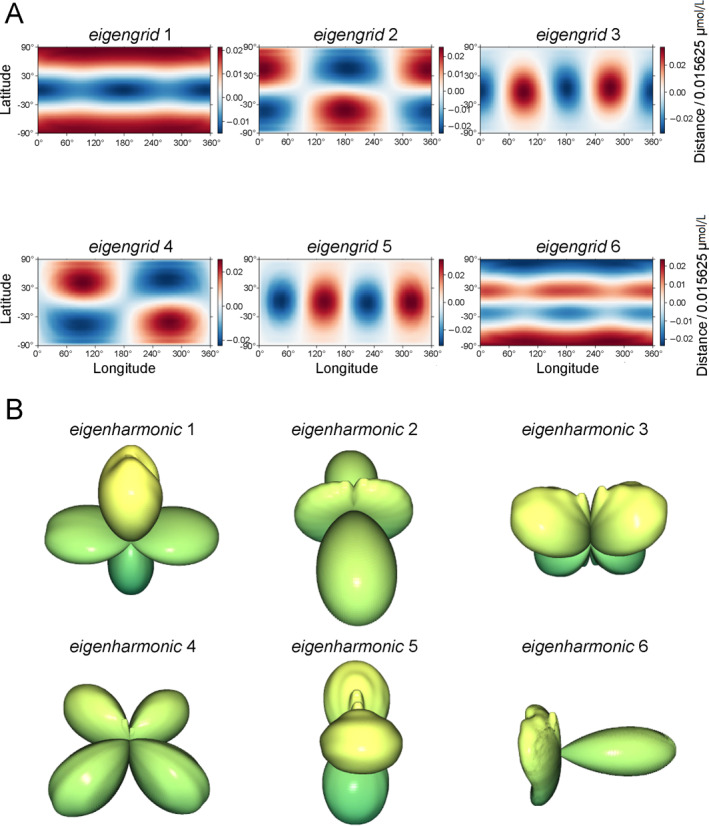
The qualitative demonstrations of *eigengrid* and *eigenharmonic* in dynamic lineage. (A) *eigengrid* components analysis: the first six *eigengrid components*, with their importance, 27%, 11.8%, 10.4%, 9.5%, 7.85%, 4%, respectively. And their cumulative contribution is 71.35%. *eigengrid* component 1, capturing 27% of shape information being considered most informative. Red represents protrusions and blue represents depression, relative to the spherical surface. (B) *eigenharmonic* components analysis: the first six *eigenharmonic* components in 3D view, and details of their importance (17.2%, 14.3%, 11.9%, 11.2%, 9.7%, 2.9%). The calculation methods and *eigengrid* and *eigenharmonic* are detailed in the supplementary files.

PCA is a widely used statistical learning method. It can be considered as a kind of transformation reducing the dimension of features and you can view the actual components (Figure 6). When there are *N* cells in the dataset, the principal components (eigenvectors of covariance matrix) are derived as *eigengrid* or *eigenharmonic* $P=\left[\vec{p_{1}},\vec{p_{2}},\ldots ,\vec{p_{K}}\right]$, where *K* is the dimension used in PCA, the dimension of the principal coefficient vector (*eigengrid* and *eigenharmonic*), and $\vec{p_{i}}\in R^{d}$, while $d={\left(L+1\right)}^{2}$. For a transformed shape, an *eigengrid* or *eigenharmonic* weight vector $\vec{FP_{i}}$, i−th cell in 378,434 cells, a 2D grid or SPHARM coefficient low dimensional transformation based on *K* principal components (eigenvectors) would be $\vec{FP_{i}}={\left(P\right)}^{-1}\cdot \left(\vec{F_{i}}-\vec{\mu }\right)$. While $\vec{FP_{i}}\in R^{K}$, $\vec{F_{i}}$ is the vector representing a single cell before PCA (2D grid or SPHARM coefficients) and $\vec{\mu }\in R^{d}$, $\vec{\mu }$ is the mean vector of all cells. PCA is utilized to approximate the morphological features, which will lead to more errors for smaller *K*. Combining 2D spherical matrix or SPHARM coefficient matrix with principal component analysis, the static features of all cells, the *eigengrid* or *eigenharmonic* weight array ***F***, is generated as follows:

$$F=\left[\vec{f_{1}},\vec{f_{1}},\vec{f_{2}},\ldots ,\vec{f_{N-1}},\vec{f_{N}}\right].$$

This equation represents the whole dataset. For convenience, these two feature vectors are termed *eigengrid* and *eigenharmonic* as well (the full name should be *eigengrid weight vector* and *eigenharmonic weight vector)*. Using PCA, 2D spherical mapping and SPHARM transform are applied to every cell during embryogenesis, giving the low dimensional non‐linear representation ($\vec{FP_{i}}$, i∈N) for all 3D cell shape based on *eigengrid* or *eigenharmonic* (Figure 6A,B). Thanks to that the original cell shape could be reconstructed from *eigengrid* and *eigenharmonic*, we can find the actual shape deformations and the convex outward and concave directions for *eigengrid* 1–6, and *eigenharmonic* 1–6, respectively. *eigenspecturm* is generated via the same process as *eigengrid* and *eigenharmonic* based on $F_{\text{spectrum}}\left(l\right)$ so the generation process of it is omitted.

### 5.3 Static and dynamic shape features generation

During the lifespan cycle of a cell, the cell changes and moves within a reasonable deformation range (it should not change drastically at an interval of frame). Feature vectors ($\vec{f_{i}}$ and $F_{\text{spectrum}}\left(l\right)$) extracted from cell shape at the single time point are the static features $f_{\text{static}}$ in 3DCSQ, which contains cell shape information at that moment rather than the information of cell deformation and migration. To observe and discover the patterns on the cell lineage clearly, a cell during its cell cycle, if quantified as one value, is easier to be recognized. Another thing is that a lot of cells after 100 min (80 frames) during embryogenesis were removed in data. Because of the low qualify of 2D image slices of *C. elegans* in vivo, the failed segmentation and discontinuity of cellular changes appear frequently. To conquer these two issues, during the cell cycle, dynamic feature vectors are proposed for each cell to quantify the same cell’s movement, growth, and deformation before it divides which contains the time information from its appearance to its division. The dynamic feature vector is $f_{\text{dynamic}}$ derived from the static feature vectors of the same cell in a cell cycle:

$$f_{\text{dynamic}}\left(cell\right)=Avg_{N_{\text{static}}\left(cell\right)}f_{\text{static}}.$$

$N_{\text{dynamic}}\left(cell\right)$ represents the collections of a cell at different time points during its cell cycle. 3DCSQ compares static features and dynamic features of cells in section results. There are three static feature vectors and three dynamic feature vectors derived from the static feature vector equation above.

### 5.4 Cell shape analysis and visualization

3DCSQ provides three feature vectors representing cell shape. This section states how to manipulate the feature vectors. Integrated PCA can reduce noise by transforming the feature vector on the direction with the largest variance. With proposed *eigengrid* and *eigenharmonic*, the cell lineage analysis is more easily conducted via plotting and recognizing the major shape changes during cell development.

A 2D spherical grid homogeneously sampled on the cell surface is applied to 378,434 cells in 17 embryos. Each 2D grid (Figure 4C) for a cell is flattened to a vector (feature vector $F_{\text{grid}}\left(\theta ,\phi \right)$). These vectors for all cells are concatenated to an array *A*. By PCA, array *A* would be transformed to another feature array and a 2D spherical grid is transformed to a vector of weights of *eigengrid* obtained by orthonormal matrix Q and an upper triangular matrix R decomposition. A total of 96 components were applied with only the first six components analyzable due to their high explained variance ratio. The first six *eigengrid* are shown in Figure 6B. The SPAHRM coefficient vector (the weights of spherical harmonics), visualized in Figure 5, is flattened to a vector and then for all feature vectors $F_{\text{sp}h\text{arm}}\left(l,m\right)$ were concatenated. Since that 3DCSQ transforms SPHARM to *eigenharmonic* coefficients, this vector (principal component weights) allows cell shape observation on the cell lineage tree via *eigenharmonic*. The first *eigengrid* and *eigenharmonic* contain most cell shape information, so the characteristics of this featured shape also exist in the corresponding cell shape. The reconstructed shapes of the first six *eigenharmonic* are shown in Figure 6B.

For visualizing lineage cell shape to get a continuous cell lineage tree to reveal cell shape patterns, average cell lineage tree (average embryo) with cell fate is shown in Figure 3A. The tree is the average cell lineage tree (the average cell cycle, from cell appearance to cell division or death, of 17 embryos). In qualitative detail, the cell lineage tree is the average cell shape of the same cells of 17 embryos at this time point (Figure 2A). The lifespan cell cycle in average cell lineage tree (among individuals) is defined as follows:

$$Range\left(Cell\right)=1/\left(n\left[\sum_{i=1}^{n}TP_{\text{CellStart}},\sum_{i=1}^{n}TP_{\text{CellEnd}}\right]\right).$$

The n is the number of the individuals (embryos). The average cell lineage tree can be established via average cell between individuals and traced cell fate which is known. The stereotypy of *C. elegans* (cell shape reproducible phenomenon), in Section *Shape Reproducibility of C. elegans*, is not obvious in embryos’ cell lineage tree by 350‐cell stage (around 220 min) because of the loss and erroneous segmentation of single cells after 150 min in embryos. To robustly analyze cell shape quantification results with cell lineage, 3DCSQ uses the weight of the first *eigengrid* (static features) as the quantification result of the cell shape; this weight contains 27% variance information, its level of changes in cell lineage tree can represent the changes of corresponding cell. With calculating $f_{\text{static}}\left(cell,tp\right)$, N(cell,tp) is integrated as follows:

$$f_{\text{static}}\left(cell,tp\right)=Avg_{N\left(cell,tp\right)}f,$$

where tp is the time point this cell appears among embryos. Using $f_{\text{static}}$ in the average cell lineage tree to do patterns analyses allows the cell shape reproducibility and recognition more effective and practical. The average cell lineage tree combined 17 embryos’ information, which is more universal and convincing than using single embryo. The results and discoveries are demonstrated in section results.

### 5.5 Feature vector clustering

Different clustering methods are applied in these three feature vectors. 3DCSQ compares K‐means, DBSCAN (density based spatial clustering), and hierarchical clustering (ward, average maximum, and single linkage method) algorithms on static or dynamic *eigengrid*, *eigenharmonic,* and eigenspectrum of every cell. To compare the clustering performance of feature vectors during embryogenesis, the silhouette coefficient is calculated using the mean intra‐cluster distance and the mean nearest‐cluster distance for each sample. The value is between −1 and 1. The best is 1 and the worst is −1, while values near 0 indicate overlapping clusters. The application and experiment results are showed in section esults.

## AUTHOR CONTRIBUTION

**Zelin Li:** Formal analysis; Software; Investigation; Validation; Methodology; Visualization; Writing – original draft. **Zhaoke Huang:** Methodology; Validation; Writing – review & editing. **Jianfeng Cao:** Investigation; Methodology; Software. **Guoye Guan:** Supervision; Visualization; Writing – review & editing. **Zhongying Zhao:** Resources; Data curation. **Hong Yan:** Conceptualization; Funding acquisition; Project administration; Supervision; Writing – review & editing.

## CONFLICT OF INTEREST STATEMENT

The authors declare no conflicts of interest.

## ETHICS STATEMENT

No additional ethics statement should be disclosed in this study.

## Supporting information

Supplementary Material

Table S1

Table S2

Table S3

Table S4

## Data Availability

Code is available from Github website (chiellini/3DCSQ). More data could be obtained upon request.
