# Supplementary material for: An effective method for quantification, visualization, and analysis of 3D cell shape during early embryogenesis
Source: Quant Biol. 2024 Dec 20;13(1):e83. doi: 10.1002/qub2.83 (PMC12806067; doi:10.1002/qub2.83)
Supplement: Supplementary file 1 — Supplementary Material [file QUB2-13-e83-s001.pdf]

# SUPPLEMENTARY MATERIALS

## of 3DCSQ: An effective study for quantification, visualization and analysis of 3D cell shape during early embryogenesis

Zelin Li, Zhaoke Huang, Jianfeng Cao, Guoye Guan, Zhongying Zhao, Hong Yan

Corresponding authors: zelinli6-c@my.cityu.edu.hk (Zelin Li) and guanguoye@gmail.com (Guoye Guan)

- "eigengrid weights" - *eigengrid* (*eigengrid* weight vector*)*
  - The folder named "eigengrid weights" contains the weights of *eigengrids* in all cells in every embryo, which is also the quantified features of cell shape from 3DCSQ.
- "eigenharmonic weights" - *eigenharmonic* (*eigenharmonic* weight vector*)*
  - The folder named "eigenharmonic weights" contains the weights of *eigenharmonics* in all cells in every embryo, which is also the quantified features of cell shape from 3DCSQ.
- "eigenspectrum coefficient" - *eigenspectrum* (*eigenspectrum* weight vector)
  - The folder named "eigenspectrum coefficient" contains the weights of PCA transformed rotation-invariant *energy spectrum* in all cells in every embryos, which is also the most effective quantified features of cell shape from 3DCSQ.

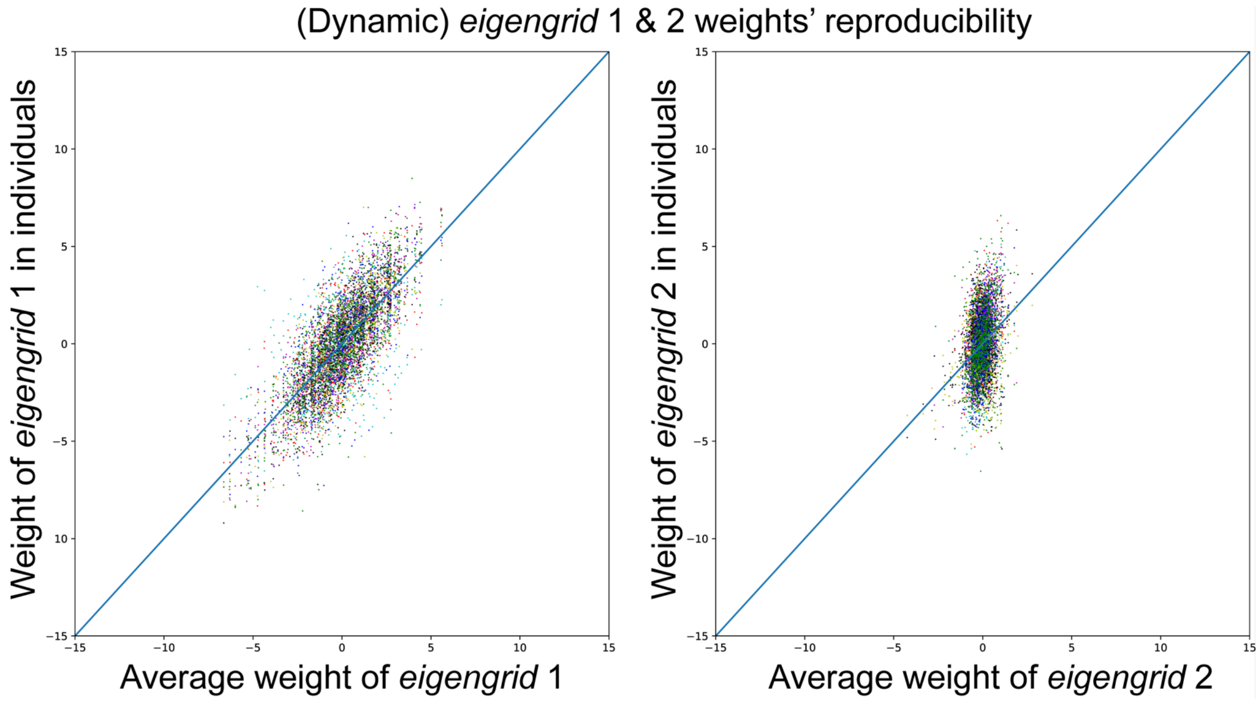


- Figure S1 – the cell shape reproducibility using *eigengrid* for 8 uncompressed wild-type embryos
  - *eigengrid* 1 showcases the linear relationship with the average value (cell shape reproducibility among embryos/individuals)
  - *eigengrid* 2 show no reproducibility because of the unrecognized deformations

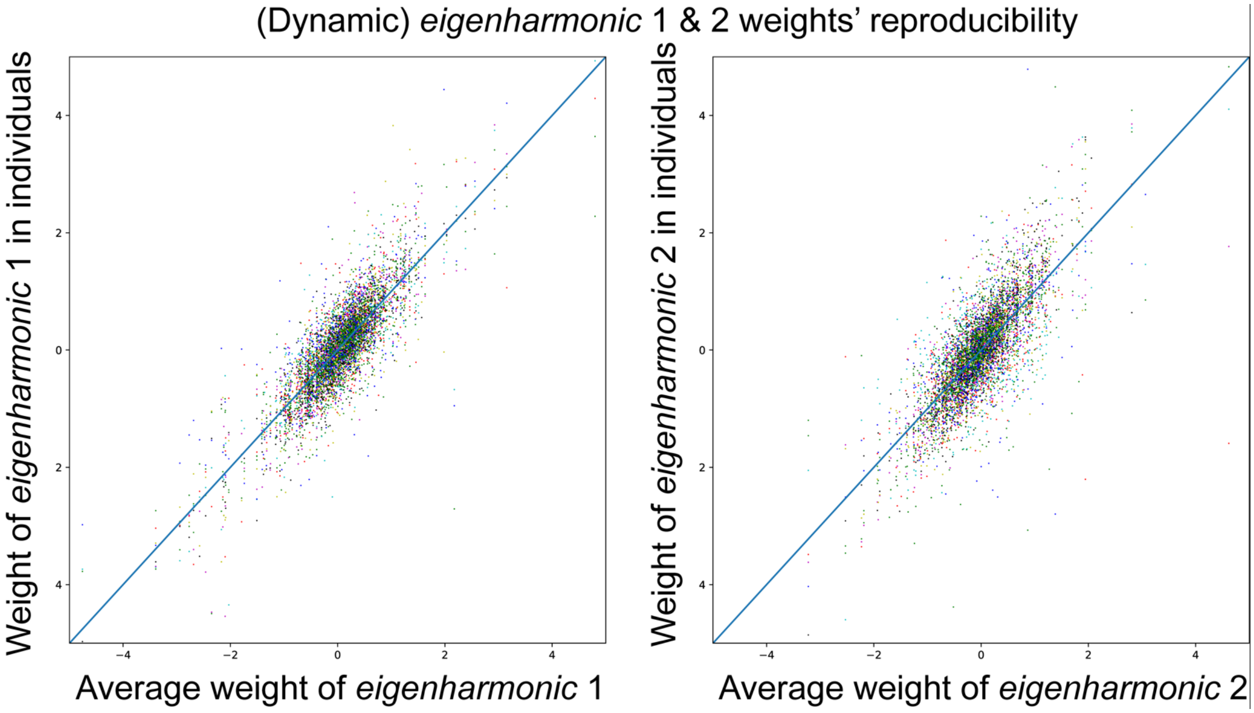


- Figure S2 - the cell shape reproducibility using *eigenharmonic* for 8 uncompressed wild-type embryos
  - *eigenharmonic* 1 and 2 showcases the linear relationship with the average value (cell shape reproducibility among embryos/individuals)

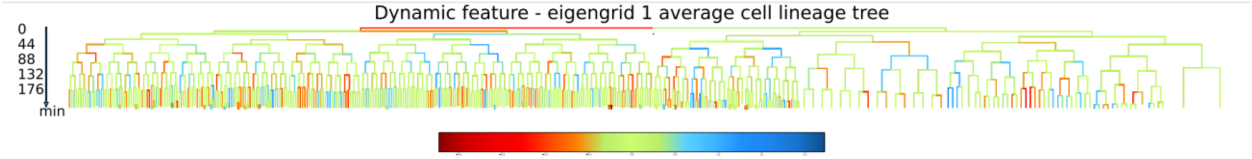


- Figure S3 – the lineage analysis of *eigengrid* 1 of 8 uncompressed embryos
  - The figure showcases the lineage tree analysis with *eigengrid* 1

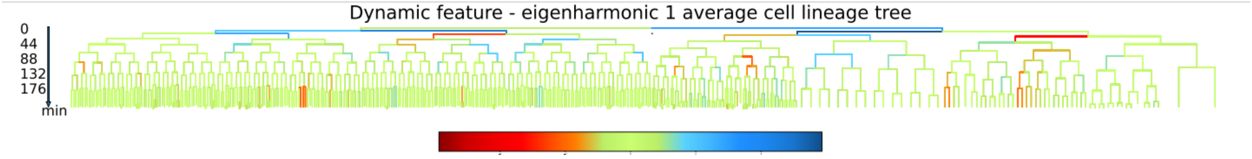


- Figure S4 – the lineage analysis of *eigenharmonic* 1 of 8 uncompressed embryos
  - The figure showcases the lineage tree analysis with *eigenharmonic* 1

- Table S1 – the dynamic *eigengrid* (for lineage analysis)
  - The table shows the dynamic *eigengrid* for every cell.
- Table S2 – the dynamic *eigenharmonic* (for lineage analysis)
  - The table shows the dynamic *eigenharmonic* for every cell.
- Table S3 - the components of *eigengrid* (for shape reconstruction)

  - File named “Table S3.csv” is the principle component values (PCA) from spherical grid, also termed as *eigengrid*.

- Table S4 - the components of *eigenharmonic* (for shape reconstruction)
  - File named “Table S4.csv” is the principle component values (PCA) from spherical harmonic, also termed as *eigenharmonic*.
